# Anatomically Defined Motor Phenotypes Reveal Dissociated Trajectories of Progression, Neurofilament Profiles, and Survival in ALS

**DOI:** 10.1111/ene.70798

**Published:** 2026-10-27

**Authors:** Thomas Meyer, Torsten Grehl, Peggy Schumann, Ute Weyen, Annekathrin Rödiger, Uta Smesny, Benjamin Ilse, Patrick Weydt, Sarah Bernsen, Rachel Fabian, Bettina Göricke, Susanne Petri, Rea Lumi, Bogdan Bjelica, Julian Großkreutz, Hakan Cetin, Omar Keritam, Friedrich Haimberger, Catharina Korsukewitz, Wolfgang Löscher, Zaharias Kohl, Martin Regensburger, Petra Baum, Moritz Metelmann, Dagmar Kettemann, Jenny Norden, Robert Steinbach, Péter Körtvélyessy, Paul Lingor, Robert McFarlane, Christoph Münch, Matthias Boentert, André Maier

**Affiliations:** ^1^ Department of Neurology, Center for ALS and Other Motor Neuron Disorders Charité – Universitätsmedizin Berlin, Corporate Member of Freie Universität Berlin, Humboldt‐Universität Zu Berlin, and Berlin Institute of Health Berlin Germany; ^2^ APST Research Berlin Germany; ^3^ Department of Neurology, Center for ALS and Other Motor Neuron Disorders Alfried Krupp Krankenhaus Essen Germany; ^4^ Department of Neurology, BG University Hospital Bergmannsheil Ruhr University Bochum Bochum Germany; ^5^ Department of Neurology Jena University Hospital Jena Germany; ^6^ ZSE, Zentrum für Seltene Erkrankungen Jena University Hospital Jena Germany; ^7^ Department for Neuromuscular Disorders Bonn University Bonn Germany; ^8^ DZNE, Deutsches Zentrum für Neurodegenerative Erkrankungen, Research Site Bonn Germany; ^9^ Department of Neurology Universitätsmedizin Göttingen Göttingen Germany; ^10^ Department of Neurology Hannover Medical School Hannover Germany; ^11^ Department of Neurology Diakovere Henriettenstift and Friederikenstift Hannover Germany; ^12^ PRACTIS Clinician Scientist Program, Dean's Office for Academic Career Development Hannover Medical School Hannover Germany; ^13^ Department of Neurology Universitätsmedizin Schleswig‐Holstein Lübeck Germany; ^14^ Department of Neurology Medical University of Vienna Vienna Austria; ^15^ Comprehensive Center for Clinical Neurosciences and Mental Health Medical University of Vienna Vienna Austria; ^16^ Department of Neurology Münster University Hospital Münster Germany; ^17^ Department of Neurology Medical University Innsbruck Innsbruck Austria; ^18^ Department of Neurology University of Regensburg Regensburg Germany; ^19^ Department of Molecular Neurology Friedrich‐Alexander‐University Erlangen‐Nürnberg Erlangen Germany; ^20^ Center for Rare Diseases Erlangen (ZSEER) University Hospital Erlangen Erlangen Germany; ^21^ Department of Neurology University Hospital Leipzig Leipzig Germany; ^22^ Department of Neurology, Klinikum Rechts der Isar Technical University of Munich Munich Germany; ^23^ DZNE, Deutsches Zentrum für Neurodegenerative Erkrankungen, Research Site Munich Germany; ^24^ Academic Unit of Neurology, Trinity Biomedical Sciences Institute, School of Medicine Trinity College Dublin, The University of Dublin Dublin Ireland; ^25^ Neuromedizinisches Zentrum Klinikum Osnabrück Osnabrück Germany

**Keywords:** amyotrophic lateral sclerosis, motor phenotypes, neurofilament light chain, propagation, survival

## Abstract

**Background:**

Clinical heterogeneity in amyotrophic lateral sclerosis (ALS) may reflect structured disease trajectories. This study investigated whether anatomically defined motor phenotypes capture reproducible patterns associated with progression, serum neurofilament light chain (NfL), and survival.

**Methods:**

In a multicenter cohort of 1307 ALS patients, motor phenotypes were classified by region of onset, timing of propagation to a vertically distant body region (early ≤ 12 months vs. later), and the degree of upper (UMN) and lower motor neuron (LMN) dysfunction. Intercenter consistency was assessed using the Friedman test and Kendall's W. Associations with ALS progression rate (ALSPR), NfL, and survival were analyzed using group comparisons and Cox regression models.

**Results:**

Phenotype classification was consistent across centers (Kendall's W 0.75–0.89). Onset phenotypes were significantly associated with ALSPR, NfL, and survival (*p* < 0.001). Trunk onset showed the lowest log(NfL/ALSPR), indicating dissociation between rapid progression and relative NfL elevation. Early propagation was the strongest marker of disease aggressiveness, with higher ALSPR, elevated NfL, and reduced survival (HR 2.93, *p* < 0.001). In early propagation, NfL disproportionally increased with progression indicating a relative uncoupling of NfL from functional decline. Phenotypes with balanced UMN/LMN and predominant LMN involvement were associated with shorter survival (HR 2.51, *p* < 0.001).

**Conclusions:**

Anatomically defined motor phenotypes identify reproducible ALS trajectories with distinct clinical and neurofilament profiles. Early propagation provides an actionable risk marker of disease aggressiveness, while combined upper and lower motor neuron dysfunction or lower motor neuron predominance is prognostically unfavorable. These findings support integrating structured phenotyping into prognostic models and clinical trial stratification.

## Graphical abstract

This multicenter study shows that anatomically defined ALS motor phenotypes capture reproducible differences in the ALS progression rate, serum neurofilament light chain (NfL), and survival. Early propagation provides an actionable risk marker of disease aggressiveness, with faster progression, higher NfL, and reduced survival (HR 2.93, *p* < 0.001), while combined upper and lower motor neuron dysfunction or lower motor neuron predominance was also prognostically unfavorable. These findings support integrating structured phenotyping into prognostic models and clinical trial stratification.

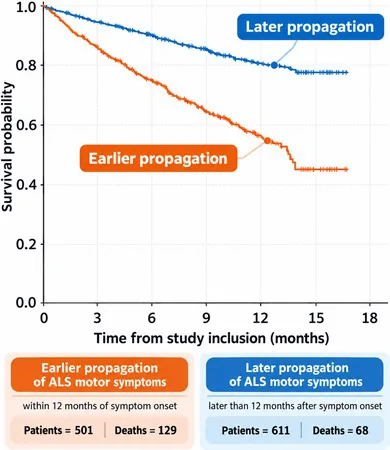

## 1 Introduction

In amyotrophic lateral sclerosis (ALS), distinct motor phenotypes are a fundamental hallmark of the disease and have been associated with differences in disease progression, neurofilament light chain (NfL) levels, and survival [1, 2, 3, 4, 5, 6, 7, 8]. From a clinical perspective, phenotype classification supports prognostic assessment and may guide symptomatic management and care at an individual level. Furthermore, ALS phenotypic data have important implications for both stratification and inclusion in clinical trials. This is based on the assumption that distinct ALS variants progress differently and may therefore influence the response to investigational therapies [9, 10].

Only recently, a classification of ALS motor phenotypes—the ALS‐OPM classification—was introduced [11, 12]. Briefly, this framework is based on three anatomical determinants: the region of onset, the temporal pattern of propagation of motor symptoms, and the degree of upper motor neuron (UMN) and/or lower motor neuron (LMN) dysfunction [13, 14, 15, 16, 17]. ALS is characterized by focal disease onset followed by a spatially structured propagation of motor symptoms to other body regions, reflecting neuroanatomic spread of pathology along contiguous and network‐connected motor pathways rather than unspecific temporal progression. This concept allows the temporal pattern of propagation to vertically distant body regions to be interpreted as a clinically accessible measure of spreading dynamics across the motor neuraxis [13, 14, 15, 16, 17]. This improved modeling of motor phenotypes should also be regarded alongside established methods of determining disease progression [18]. Previous reports have suggested a correlation between distinct ALS phenotypes and ALS progression (ALSPR), defined as the monthly change in the ALS Functional Rating Scale–Revised (ALSFRS‐R) since symptom onset [6, 7, 18]. ALSPR is widely used to characterize disease trajectory in both research and clinical practice [19, 20]. Consequently, most clinical trial protocols have incorporated ALSPR, typically operationalized as the change in ALSFRS‐R from baseline, as a key outcome measure [9, 10, 19].

Neurofilament light chain has emerged as a robust biomarker of axonal damage and a marker of disease activity and prognosis in ALS [5, 6, 7, 8, 21]. However, earlier studies investigating ALS phenotypes have demonstrated that differences in NfL levels across phenotypes are not entirely explained by differences in disease progression [7]. This observation supports the concept that anatomical determinants of ALS phenotypes may contribute distinct profiles of NfL elevation [5, 7]. To quantify NfL elevation relative to disease progression, the NfL–ALSPR index—defined as the log‐transformed ratio of serum NfL to ALSPR (log(NfL/ALSPR))—was recently introduced [22].

To evaluate the ALS‐OPM classification in relation to key outcome parameters in ALS, data from a multicenter observational study were analyzed. The present investigation focused on the association between ALS‐OPM phenotypes, ALSPR, serum NfL, and survival.

## 2 Methods

### 2.1 Study Design

This cross‐sectional analysis was based on data from a prospective, multicenter, longitudinal observational study. The investigation was reported according to the STROBE criteria [23].

### 2.2 Participants and Definition of Cohorts

Participants met the diagnostic criteria of ALS referring to the Gold Coast criteria [24]. ALS‐OPM classification as described below was made by experienced neurologists at the participating study centers. A definition of studied cohorts and subgroups is provided in Figure 1.

**FIGURE 1:**
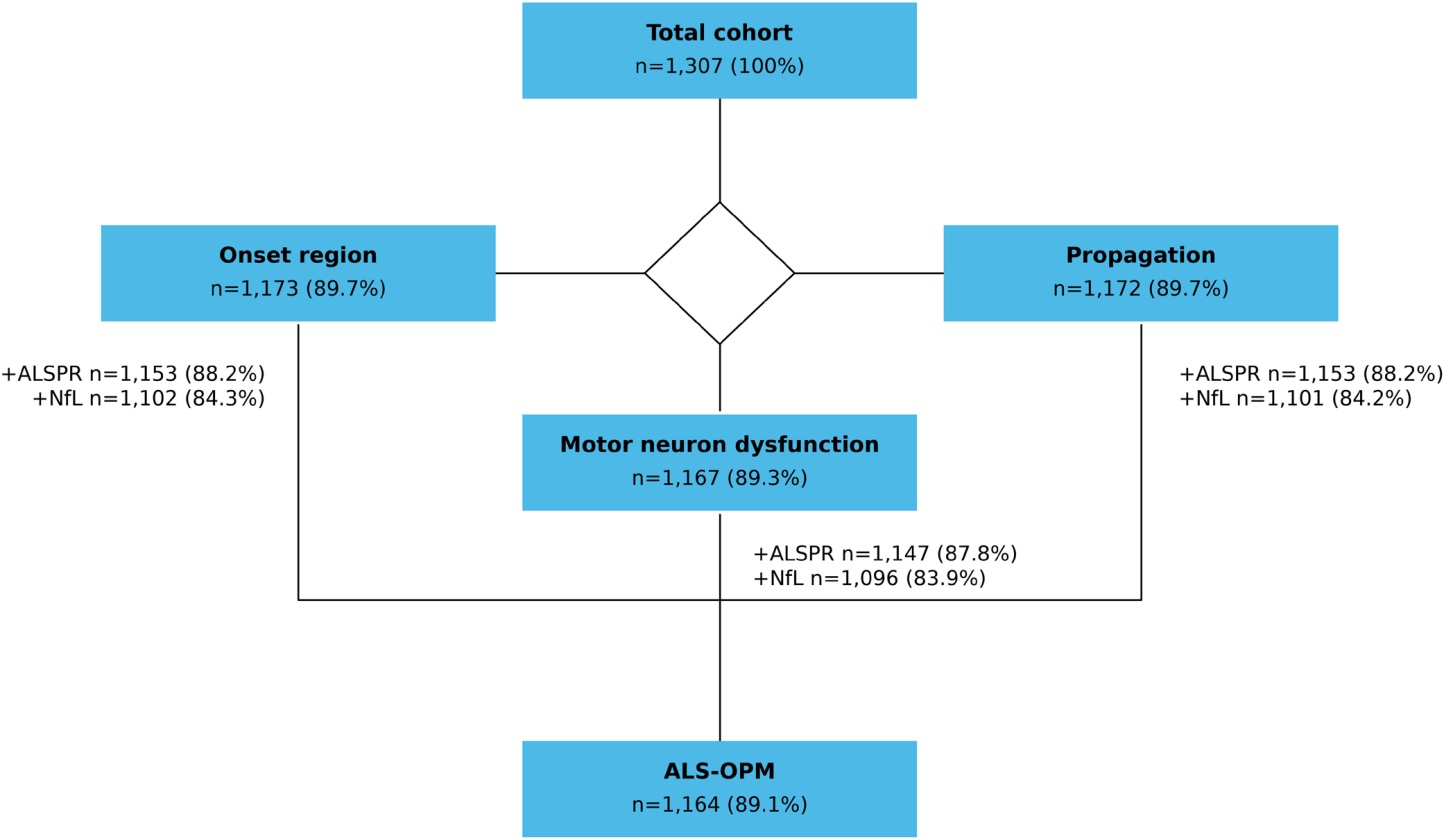
Studied ALS cohort and data availability. For phenotyping, three anatomic determinants were distinguished: onset region of motor symptoms; propagation of motor symptoms; motor neuron dysfunction. ALS, amyotrophic lateral sclerosis; ALSPR, ALS progression; OPM, ALS‐OPM motor phenotype classification *n* = number of patients; NfL, neurofilament light chain.

### 2.3 Setting

#### 2.3.1 Recruitment

Following informed consent, patients were recruited at 15 multidisciplinary ALS centers in Germany and Austria between April and December 2025.

#### 2.3.2 Data Collection

ALS‐OPM phenotypes (version 3.1) were classified by a neurologist using the respective clinical research form and a guidance document supporting ALS‐OPM assessment [11]. During all clinical visits, an assessment of the ALSFRS‐R‐SE (version 1.0) was performed [25]. Additional ALSFRS‐R‐SE data were captured by remote digital assessment of the ALSFRS‐R‐SE using a mobile application (“ALS App”) [26, 27].

#### 2.3.3 NfL Analysis

Serum samples for NfL analysis were collected, centrifuged, aliquoted, and shipped to the ALS center in Berlin (Germany), where the core facility for serum NfL analysis was located [6, 7]. Measurement of NfL concentration was realized with the single molecule array (SIMOA) technology (HD‐X instrument, Quanterix Inc., USA) using the commercially available NfL advantage kit.

### 2.4 Protocol Approvals and Registrations

The multicenter study protocol was ethically approved by the ethics commission of Charité—Universitätsmedizin Berlin under numbers EA2/168/20. A signed informed consent form was obtained from all study participants.

### 2.5 Variables

#### 2.5.1 Demographic and Clinical Characteristics

The following demographic and clinical characteristics were collected (Table 1): age at disease onset, sex, disease duration (number of months between disease onset and beginning of observation period), and survival (number of months between disease onset and date of death).

**TABLE 1:** Clinical characteristics, neurofilament light chain and survival in ALS‐OPM phenotypes.

	Patient number (*n*)	Age at symptom onset (years)	Duration (months)	Male/female	ALSFRS‐R	ALSPR	NfL (pg/mL)	NfL (log) index	Deceased	Survival from symptom onset (months)
Total	1307	60.7 ± 11.7	32.4 (16.9–67.0)	772 (59.1%) 535 (40.9%)	33 (26–39)	0.42 (0.21–0.78)	44.2 (25.1–78.7)	4.72 ± 0.81	218 (16.7%)	39.8 (24.0–74.1)
*Onset region phenotypes*										
O1	331 (28.2%)	64.9 ± 10.2	23.1 (13.7–44.5)	139 (42.0%) 192 (58.0%)	33 (26–40)	0.54 (0.28–0.92)	69.8 (36.4–103.0)	4.82 ± 0.77	79 (23.9%)	30.8 (20.0–51.6)
O2d	285 (24.3%)	57.2 ± 12.5	37.8 (18.6–71.0)	191 (67.0%) 94 (33.0%)	32 (24–40)	0.40 (0.20–0.69)	42.2 (22.7–72.9)	4.70 ± 0.81	47 (16.5%)	45.8 (25.6–78.1)
O2p	95 (8.1%)	61.4 ± 10.9	35.4 (21.1–54.9)	75 (78.9%) 20 (21.1%)	34 (26–42)	0.34 (0.18–0.60)	29.9 (19.2–52.9)	4.58 ± 0.91	12 (12.6%)	43.1 (28.3–65.8)
O3r	25 (2.1%)	67.5 ± 8.6	18.8 (12.2–27.6)	20 (80.0%) 5 (20.0%)	27 (21–35)	0.98 (0.74–1.49)	48.6 (30.5–84.3)	3.98 ± 0.56	7 (28.0%)	25.5 (14.1–32.9)
O3a	14 (1.2%)	69.4 ± 8.5	23.0 (14.8–27.5)	12 (85.7%) 2 (14.3%)	28 (23–38)	0.67 (0.51–1.23)	46.2 (22.4–81.8)	4.07 ± 0.66	5 (35.7%)	28.5 (21.0–38.4)
O4d	263 (22.4%)	59.1 ± 11.0	37.5 (18.5–72.5)	155 (58.9%) 108 (41.1%)	34 (28–39)	0.39 (0.21–0.69)	43.4 (25.7–67.8)	4.73 ± 0.80	35 (13.3%)	44.6 (26.4–81.4)
O4p	160 (13.6%)	59.2 ± 12.6	53.9 (26.1–106.7)	103 (64.4%) 57 (35.6%)	33 (28–38)	0.26 (0.13–0.48)	27.5 (17.5–57.5)	4.73 ± 0.77	16 (10.0%)	63.1 (31.7–113.6)
*Propagation phenotypes*										
PE	509 (43.4%)	61.8 ± 12.1	20.3 (12.2–36.2)	299 (58.7%) 210 (41.3%)	32 (24–37)	0.73 (0.44–1.19)	61.1 (37.6–98.4)	4.47 ± 0.73	129 (25.3%)	27.2 (18.1–43.7)
PL	615 (52.5%)	59.9 ± 11.4	49.2 (30.0–90.5)	365 (59.3%) 250 (40.7%)	34 (27–40)	0.26 (0.14–0.46)	32.5 (19.0–56.9)	4.91 ± 0.80	68 (11.1%)	57.0 (37.1–97.8)
PN	48 (4.1%)	60.3 ± 12.5	10.4 (8.4–15.1)	32 (66.7%) 16 (33.3%)	42 (38–45)	0.48 (0.27–0.84)	63.2 (33.6–103.3)	4.74 ± 0.92	2 (4.2%)	19.0 (16.1–24.3)
*Motor neuron phenotypes*										
M0	497 (42.6%)	61.1 ± 12.2	24.2 (14.6–44.1)	267 (53.7%) 230 (46.3%)	34 (26–39)	0.55 (0.29–0.90)	59.4 (32.4–93.8)	4.71 ± 0.76	109 (21.9%)	31.8 (21.0–51.1)
M1d	192 (16.5%)	59.5 ± 10.1	46.5 (18.3–95.2)	108 (56.2%) 84 (43.8%)	32 (25–38)	0.34 (0.18–0.56)	37.9 (23.9–69.8)	4.80 ± 0.72	23 (12.0%)	56.7 (27.7–103.4)
M1p	74 (6.3%)	59.0 ± 10.7	54.2 (33.0–101.7)	41 (55.4%) 33 (44.6%)	36 (31–40)	0.19 (0.12–0.30)	26.1 (16.4–43.0)	4.92 ± 0.77	0 (0.0%)	65.5 (40.8–107.3)
M2d	205 (17.6%)	63.2 ± 11.1	29.6 (15.9–56.0)	138 (67.3%) 67 (32.7%)	33 (27–39)	0.47 (0.23–0.87)	43.6 (26.8–73.8)	4.68 ± 0.90	39 (19.0%)	36.4 (23.1–61.0)
M2p	162 (13.9%)	61.8 ± 11.1	46.2 (24.7–84.8)	113 (69.8%) 49 (30.2%)	33 (24–39)	0.32 (0.18–0.56)	29.2 (17.5–51.7)	4.62 ± 0.91	22 (13.6%)	55.1 (31.6–94.1)
M3	37 (3.2%)	48.0 ± 12.6	44.5 (30.7–80.2)	26 (70.3%) 11 (29.7%)	30 (16–36)	0.39 (0.23–0.66)	38.0 (22.1–54.7)	4.59 ± 0.63	5 (13.5%)	54.7 (36.8–88.0)

*Note:* Categorical variables are given as number and percentage. Continuous variables are presented as mean (±SD) or median (IQR), as appropriate. O1, head onset; O2d, distal arm onset; O2p, proximal arm onset; O3r, trunk respiratory onset; O3a, trunk axial onset; O4d, distal leg onset; O4p, proximal leg onset; PE, early propagation; PL, later propagation; PN, propagation not yet classifiable; M0, balanced upper motor neuron (UMN) and lower motor neuron (LMN) dysfunction; M1d, dominant UMN; M1p, pure UMN; M2d, dominant LMN; M2p, pure LMN; M3, dissociated LMN/UMN symptoms and signs. Survival is the observed time from symptom onset to death or individual censoring at the last date known alive.

Abbreviations: IQR, Interquartile Range (25th—75th percentile); SD, standard deviation.

#### 2.5.2 ALS‐OPM Phenotypes

ALS motor phenotypes were assessed according to the onset region, vertical propagation, and the degree of upper (UMN) and lower motor neuron (LMN) dysfunction (ALS‐OPM classification 3.1, detailed description in Figure 2) [11].

**FIGURE 2:**
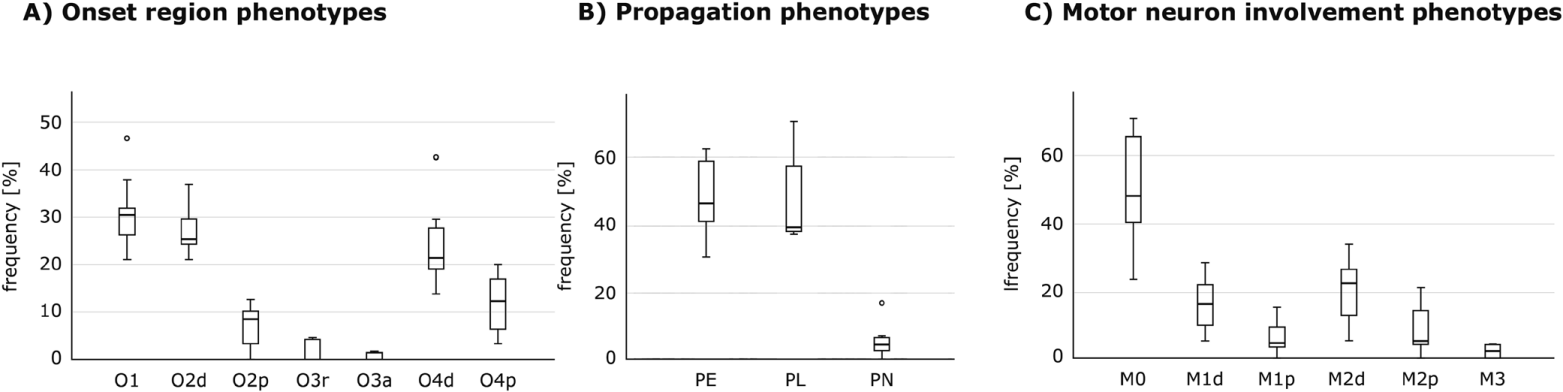
Frequency distribution of ALS‐OPM phenotypes. The line within each box represents the median across centers; boxes indicate the interquartile range; whiskers denote the range; dots represent outliers. Onset region phenotypes differentiated the site of first motor symptoms: O1, head onset; O2d, distal arm onset; O2p, proximal arm onset; O3r, trunk respiratory onset; O3a, trunk axial onset; O4d, distal leg onset; O4p, proximal leg onset. The assessment was based on the patient history. Propagation phenotypes differentiate the temporal propagation of motor symptoms from the site of onset to another, vertically distant body region: PE, early propagation (within 12 months of symptom onset); PL, later propagation (without propagation within 12 months); PN, propagation not yet classifiable as time since symptom onset is less than 12 months. The assessment was based on patient history or the presence of motor symptoms on neurological examination. Motor neuron dysfunction phenotypes differentiated the degree of UMN and/or LMN dysfunction into balanced (M0), dominant UMN (M1d), pure UMN (M1p), dominant LMN (M2d), pure LMN (M2p), and dissociated LMN/UMN symptoms and signs (M3). The assessment was based on neurological examination.

#### 2.5.3 Intercenter Consistency of ALS‐OPM Phenotypes

To evaluate intercenter consistency of ALS‐OPM phenotype frequency distributions, the frequency distribution of phenotypes in each of the centers was investigated and compared across participating study sites.

#### 2.5.4 ALS Functional Rating Scale (ALSFRS‐R)

The ALSFRS‐R is a 12‐item disease‐specific instrument for the assessment of symptoms and motor function in ALS [18]. In this study, the self‐explanatory version of the ALSFRS‐R (ALSFRS‐R‐SE) was used [25].

#### 2.5.5 ALS Progression (ALSPR)

ALSPR was measured by the monthly change of the ALSFRS‐R sum score and calculated using the following equation: (48‐ALSFRS‐R divided by disease duration (months)) [20].

#### 2.5.6 Neurofilament Light Chain in Serum (sNfL)

The measurement of sNfL concentration was given in pg/mL.

#### 2.5.7 NfL in Relation to ALSPR (NfL‐ALSPR)

To account for differences in disease progression, serum NfL was normalized to ALS progression (ALSPR) by calculating the ratio of NfL to ALSPR [22]. As both variables were strictly positive, the natural logarithm of this ratio was used to reduce skewness and improve model fit. The NfL‐ALSPR index was calculated as log(NfL/ALSPR), which is equivalent to log(NfL)—log(ALSPR).

### 2.6 Statistical Methods

Statistical analyses were performed using SPSS (Version 27.0; IBM Corp., Armonk, NY, USA) and Python (Version 3.13.14; Python Software Foundation). Graphical representations were generated using GraphPad Prism (Version 9.0.0; GraphPad Software, San Diego, CA, USA) and Python using Matplotlib (version 3.11.1). Continuous variables are reported as mean ± SD or median (IQR), as appropriate; categorical variables as *n* (%). Normality was assessed using the Shapiro–Wilk test. All tests were two‐sided with *α* = 0.05. Intercenter variability analyses were restricted to centers contributing ≥ 20 patients. Center‐specific percentages (0%–100%) of ALS‐OPM phenotypes were calculated. Differences in phenotype distributions across centers were assessed using the Friedman test. The site‐specific consistency of phenotypic classification was quantified using Kendall's coefficient of consistency (W; range 0–1); values ≥ 0.5 and ≥ 0.8 were interpreted as strong and very strong agreement, respectively. Pairwise comparisons were performed using Wilcoxon signed‐rank tests with Holm–Bonferroni correction (21 comparisons). Differences in ALSPR and log(NfL/ALSPR) across ALS‐OPM phenotypes were analyzed using one‐way ANOVA. Homogeneity of variance was assessed using Levene's test. In cases of variance heterogeneity, Welch's ANOVA with Games–Howell post hoc testing was applied; otherwise, Tukey's HSD was used. Effect sizes were estimated using *η* ^2^ and *ω* ^2^. Survival time for the Kaplan–Meier analysis was defined as the time from inclusion in the observational study to death or censoring. Patients without a documented death were right‐censored at the last date of follow‐up. Kaplan–Meier curves were compared using the log‐rank test. Cox proportional hazard regression models were used to estimate hazard ratios adjusted for age at study inclusion and sex. Kaplan–Meier estimates, log‐rank tests, and Cox proportional hazards models were calculated using the Python lifelines package (version 0.30.3). The ALS‐OPM axes represent anatomical determinants of the phenotype rather than external covariates; therefore, they were analyzed separately rather than mutually adjusted to avoid overadjustment and loss of biological interpretability. PE and PL patients with available positive NfL and ALSPR values were analyzed by HC3‐robust linear regression of log‐transformed NfL on median‐centered log(ALSPR), propagation phenotype, and their interaction, with covariate adjustment. Exponentiated estimates represent adjusted geometric NfL ratios.

## 3 Results

### 3.1 Clinical Information

The total cohort included 1307 patients with a mean age at onset of 60.7 ± 11.7 years, and 59% were male. Median disease duration at assessment was 32.4 months (IQR 16.9–67.0). The median ALSFRS‐R score was 33 (IQR 26–39), corresponding to a median ALSPR of 0.42 (IQR 0.2–0.78). Onset region, propagation, and motor neuron phenotype classifications were available for 1173 (89.7%), 1172 (89.7%), and 1167 (89.3%) patients, respectively. Complete classification across all three ALS‐OPM axes was available for 1164 patients (89.1%; Figure 1).

### 3.2 Frequency of ALS‐OPM Phenotypes

Head onset (O1) was most frequent (28.2%), followed by distal arm (O2d, 24.3%) and distal leg onset (O4d, 22.4%); proximal limb and trunk phenotypes were less common (O4p 13.6%, O2p 8.1%, O3r 2.1%, O3a 1.2%). Later and early propagation occurred in 52.5% and 43.4%, respectively; 4.1% were unclassifiable. Balanced UMN/LMN dysfunction (M0) predominated (42.6%), followed by M2d (17.6%), M1d (16.5%), M2p (13.9%), M1p (6.3%), and M3 (3.2%) (Table 1, Figure 2).

### 3.3 Intercenter Consistency of ALS‐OPM Phenotypes

Intercenter variability was significant for the onset (O; Friedman *χ* ^2^(6) = 48.05, *p* < 0.001), propagation (P; *χ* ^2^(2) = 13.56, *p* = 0.001), and motor neuron dysfunction axes (M; *χ* ^2^(5) = 34.51, *p* < 0.001). Nevertheless, classification consistency across centers was high for all three axes (Kendall's W = 0.89, 0.75, and 0.77, respectively; Table S1).

### 3.4 Clinical and Biomarker Profiles of ALS‐OPM Phenotypes

#### 3.4.1 Onset Region Phenotypes

Phenotypes differed in ALSPR and NfL (both *p* < 0.001). ALSPR was highest in O3r/O3a and lowest in O4p; O1 progressed faster than limb phenotypes. NfL was highest in O1, exceeding limb phenotypes (*p* < 0.001), but comparable in trunk onset. O3r had lowest NfL‐ALSPR index (*p* < 0.001), indicating disproportionate functional decline. Survival differed (log‐rank *χ* ^2^(6) = 35.2, *p* < 0.001; Table 1, Figures 3, 4, 5). Adjusted O1 hazard was higher versus O2p (HR = 2.02; 95% CI 1.09–3.76; *p* = 0.026), O4d (HR = 1.70, 95% CI 1.13–2.57; *p* = 0.011), and O4p (HR = 2.75, 95% CI 1.59–4.75; *p* < 0.001; Figure 6).

**FIGURE 3:**
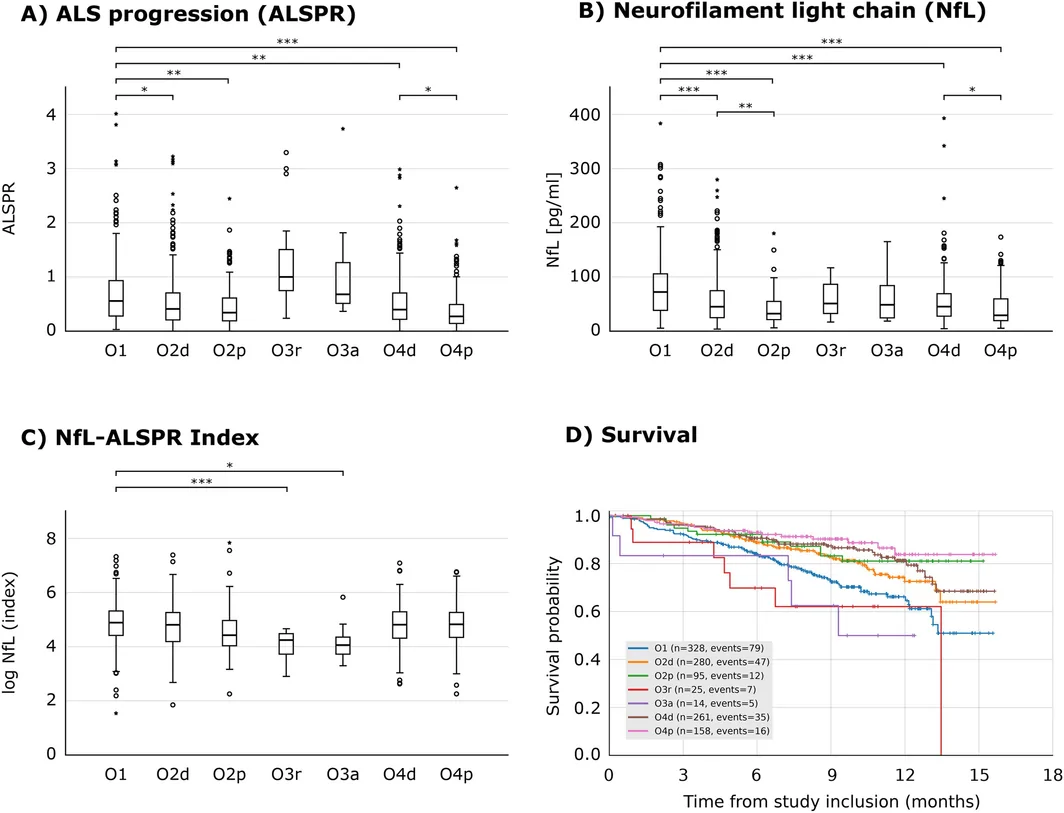
Onset region phenotypes. O1, head onset; O2d, distal arm onset; O2p, proximal arm onset; O3r, trunk respiratory onset; O3a, trunk axial onset; O4d, distal leg onset; O4p, proximal leg onset. ALS, amyotrophic lateral sclerosis; ALSPR, ALS progression; *n* = number of patients; NfL, neurofilament light chain; NfL‐ALSPR index, log‐transformed NfL‐to‐ALSPR ratio (log(NfL/ALSPR)). Asterisks denote statistical significance: *p < 0.05; **p < 0.01; ***p < 0.001.

**FIGURE 4:**
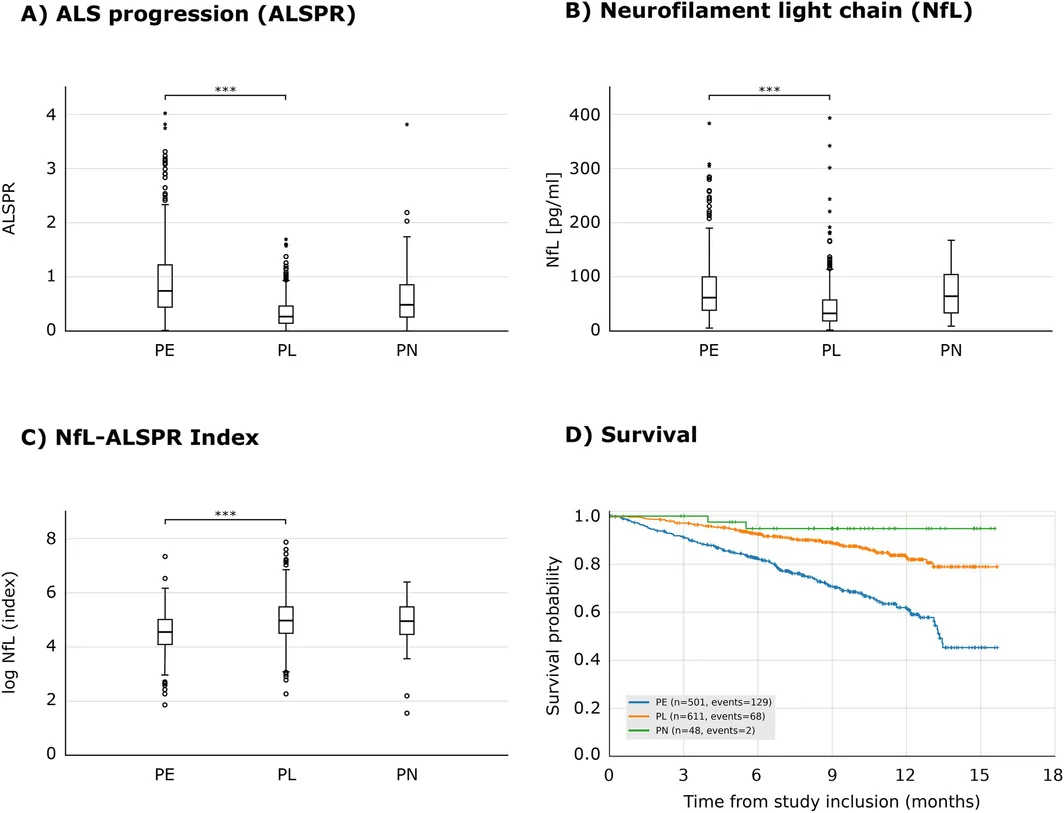
Propagation phenotypes. (PE) early propagation (within 12 months of symptom onset); PL, later propagation (without propagation within 12 months of symptom onset); PN, propagation not yet classifiable as time since symptom onset is less than 12 months. ALS, amyotrophic lateral sclerosis; ALSPR, ALS progression; *n* = number of patients; NfL, neurofilament light chain; NfL‐ALSPR index, log‐transformed NfL‐to‐ALSPR ratio (log(NfL/ALSPR)). Asterisks denote statistical significance: *p < 0.05; **p < 0.01; ***p < 0.001.

**FIGURE 5:**
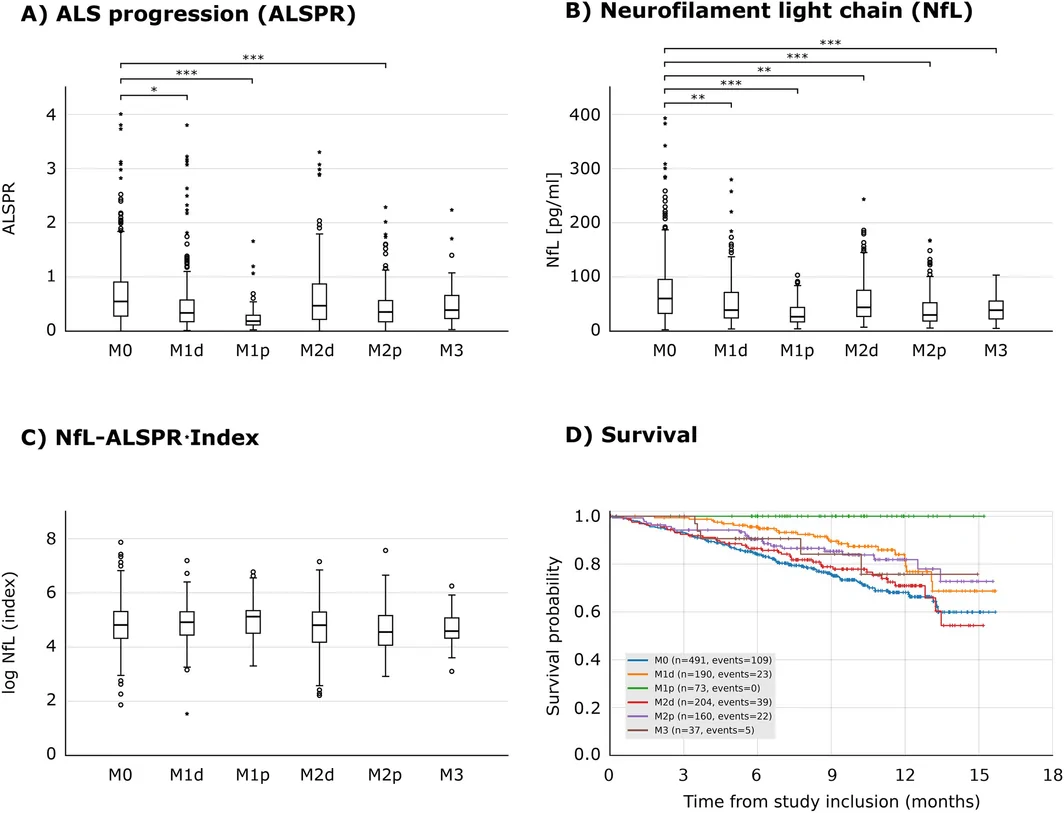
Motor neuron dysfunction phenotypes. The degree of UMN and/or LMN dysfunction was differentiated into balanced (M0), dominant UMN (M1d), pure UMN (M1p), dominant LMN (M2d), pure LMN (M2p), and dissociated LMN/UMN symptoms and signs (M3). Abbreviations: ALS, amyotrophic lateral sclerosis; ALSPR, ALS progression; *n* = number of patients; NfL, neurofilament light chain; NfL‐ALSPR index, log‐transformed NfL‐to‐ALSPR ratio (log(NfL/ALSPR)). Asterisks denote statistical significance: *p < 0.05; **p < 0.01; ***p < 0.001.

**FIGURE 6:**
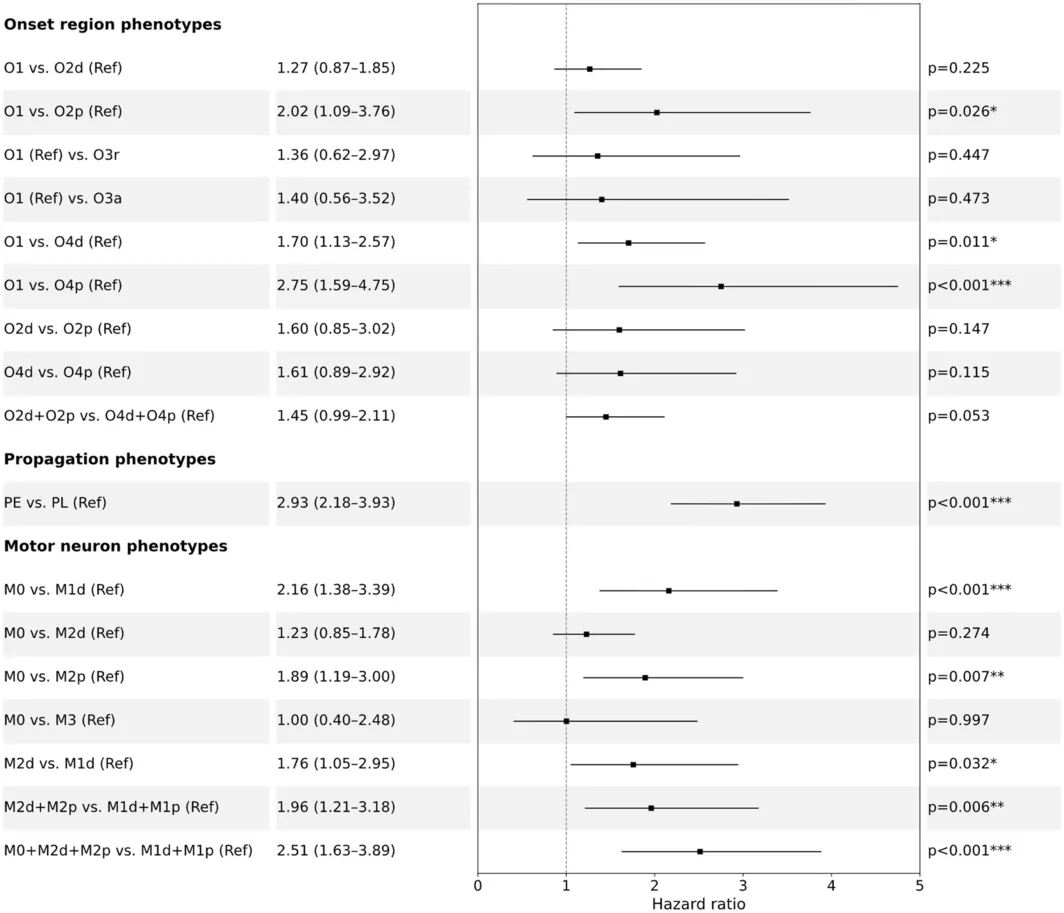
Hazard ratio (HR) to death for ALS‐OPM phenotypes. Forest plot showing hazard ratios (HRs) for death associated with ALS‐OPM phenotypes across the three anatomical axes of the ALS‐OPM classification. Hazard ratios with 95% confidence intervals were derived from Cox proportional hazards models adjusted for age at onset and sex. For onset region phenotypes, head onset (O1) served as the reference category. For motor neuron phenotypes, balanced upper and lower motor neuron involvement (M0) was used as the reference. Points represent hazard ratios and horizontal lines indicate 95% confidence intervals; the vertical dashed line denotes HR = 1. Statistically significant comparisons are indicated by asterisks. Survival time was defined from symptom onset to death or last follow‐up. Abbreviations: ALS, amyotrophic lateral sclerosis; ALSPR, ALS progression; O1, head onset; O2d, distal arm onset; O2p, proximal arm onset; O3r, trunk respiratory onset; O3a, trunk axial onset; O4d, distal leg onset; O4pm, proximal leg onset; PE, early propagation (within 12 months of symptom onset); PL, later propagation (without propagation within 12 months of symptom onset); PN, propagation not yet classifiable as time since symptom onset is less than 12 months; M0, balanced upper motor neuron (UMN) and lower motor neuron (LMN) dysfunction; M1d, dominant UMN; M1p, pure UMN; M2d, dominant LMN; M2p, pure LMN; M3, dissociated LMN/UMN symptoms and signs. Asterisks denote statistical significance: *p < 0.05; **p < 0.01; ***p < 0.001.

#### 3.4.2 Propagation Phenotypes

ALSPR differed across propagation phenotypes (*p* < 0.001), with faster progression in PE than PL. Serum NfL was also higher in PE (*p* < 0.001), whereas the NfL‐ALSPR index was lower (*p* < 0.001), indicating lower NfL elevation relative to functional decline. Survival differed markedly (log‐rank *p* < 0.001) (Table 1, Figures 3, 4, 5). After adjustment for age and sex, PE was associated with shorter survival than PL (HR 2.93, 95% CI 2.18–3.93; *p* < 0.001; Figure 6). Further analysis of the P‐axis showed that the relationship between NfL and ALS progression differed according to propagation phenotype. Faster ALS progression was predominantly associated with early propagation and higher NfL levels, whereas a smaller subgroup of faster‐progressing ALS patients exhibited later propagation. Among patients with similarly high progression rates, those with PE had higher NfL levels than those with PL (Figure 7; Table S2).

**FIGURE 7:**
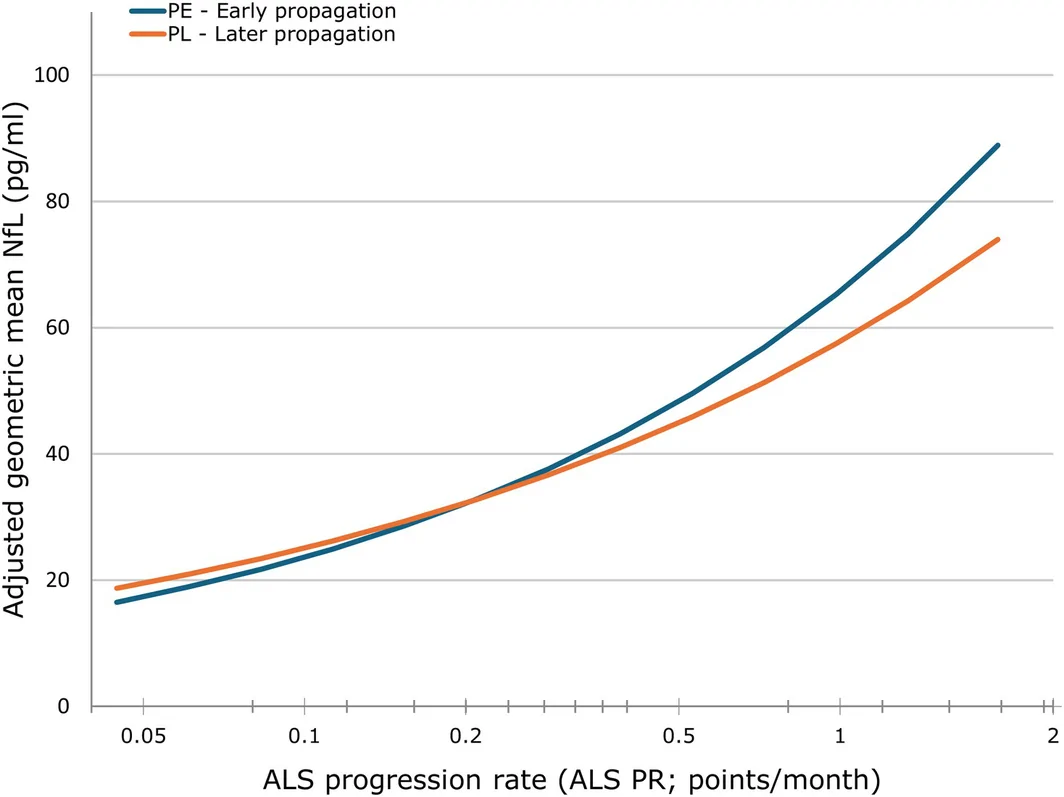
Neurofilament light chain across ALS progression rates in early and later propagation phenotypes. Adjusted geometric mean neurofilament light chain (NfL) concentrations are plotted against the ALS progression rate (ALS PR; points/month, logarithmic scale) for patients with early propagation (PE; blue) and later propagation (PL; orange).

#### 3.4.3 Motor Neuron Dysfunction Phenotypes

ALSPR and NfL differed across M phenotypes (both *p* < 0.001). M0 showed the fastest progression and highest NfL, whereas M1p progressed slowest; M1p/M2p and M3 had lower NfL. The NfL‐ALSPR index did not differ (*p* = 0.060). Survival differed across phenotypes (log‐rank *χ* ^2^(4) = 15.18, *p* = 0.004; Table 1, Figures 3, 4, 5). Adjusted mortality was higher in M0 than M1d (HR 2.16, 95% CI 1.38–3.39; *p* < 0.001) and M2p (HR 1.89, 95% CI 1.19–3.00; *p* = 0.007). Pooled LMN phenotypes had higher mortality than UMN phenotypes (HR 1.96, 95% CI 1.21–3.18; *p* = 0.006); M1p had no deaths (Figure 6).

## 4 Discussion

In this large multicenter cohort, the ALS‐OPM classification was applied at scale for the first time [11]. The frequency distribution of onset phenotypes was consistent with prior reports using less granular classifications [2, 3, 4, 28]. However, ALS‐OPM introduces a distal–proximal distinction for arm and leg onset, enabling more differentiated anatomical characterization. Proximal arm onset (O2p) occurred in 8.1%, closely corresponding to reported frequencies of flail‐arm syndrome [2, 3, 29]. By contrast, proximal leg onset (O4p) accounted for 13.6%, representing a substantial, underreported subgroup. Current definitions of flail‐leg syndrome are largely confined to distal leg onset [29, 30]. Future studies should therefore address O4p, which may refine existing phenotype definitions. Trunk onset phenotypes (O3r/a) were rare, at frequencies comparable to previous reports (approximately 4%) [2, 3, 31]. Respiratory trunk onset (O3r) was more frequent (2.1%), whereas axial trunk onset (O3a) was very rare (1.%). Further studies should delineate their distinct trajectories. Early propagation (PE) occurred in more than half of patients, whereas later vertical propagation (PL) occurred in 43.4%. The substantial PL proportion supports further temporal stratification for more granular prognostic assessment. The balanced UMN/LMN phenotype (M0) was most frequent but comprised less than half of patients, highlighting the relevance of predominant and pure UMN/LMN variants. Pure UMN (M1p) and pure LMN (M2p) phenotypes were more frequent than previously reported primary lateral sclerosis (PLS) and progressive muscular atrophy (PMA), respectively [2, 3, 32, 33], likely reflecting the formal 48‐month temporal criterion for PLS and PMA [32, 34]. Although rare (3.2%), the newly introduced M3 phenotype showed a distinct ALSPR and NfL profile, supporting its inclusion as a separate motor phenotype [35].

### 4.1 Intercenter Consistency

Phenotyping was performed by experienced ALS neurologists provided with written ALS‐OPM guidance but no additional formal training. Consistent phenotype frequencies across centers support the feasibility of the classification for clinical investigators. Intercenter consistency was very strong for onset phenotypes and strong for propagation and motor neuron dysfunction. The guidance document may have helped standardize clinical assessment. Differentiating balanced (M0) from predominant upper (M1d) or lower motor neuron (M2d) phenotypes can be challenging, reflecting subjective elements inherent to clinical ratings. Additionally, variations in phenotype frequencies across centers may reflect regional differences in referral patterns or genetic makeup, particularly for rare phenotypes. Overall, the high intercenter consistency demonstrates that ALS‐OPM phenotyping is reproducible and suitable for standardized application in multicenter studies and clinical trials.

### 4.2 Onset Phenotypes

Head region onset (O1) was associated with higher ALSPR and NfL levels and lower survival than limb‐onset phenotypes, consistent with previous reports [7, 8]. Earlier classifications primarily distinguished head (“bulbar/pseudobulbar”) from limb (“spinal”) onset [2, 3, 4, 28]. ALS‐OPM extends this concept by differentiating arm and leg onset and introducing a distal–proximal distinction within limb phenotypes. Significant distal–proximal differences were observed for both arm (O2d vs. O2p) and leg (O4d vs. O4p) onset, supporting this approach. Proximal arm onset (O2p) showed lower progression and NfL levels and more favorable survival, corresponding to flail arm syndrome, previously linked to slower progression and prolonged survival [2, 3, 29]. Unlike classical definitions, ALS‐OPM does not require a predefined observation period; nevertheless, proximal onset alone identified a distinct disease trajectory. In clinical trials, O2p may represent a relevant stratification variable or potential exclusion criterion to reduce population heterogeneity. Trunk onset phenotypes, particularly respiratory onset (O3r), exhibited the most rapid progression, higher NfL levels, and lowest survival probability [3, 7]. Accordingly, O3 phenotypes are clinically and prognostically relevant for patient management and trial design. To quantify NfL elevation relative to ALS progression, the NfL‐ALSPR index was applied [22]. Despite the highest ALSPR, trunk onset phenotypes showed significantly lower NfL‐ALSPR index (log(NfL/ALSPR)) values than head and limb onset phenotypes. One explanation is that respiratory trunk onset reflects early involvement of compact, functionally critical motor neuron pools, where strategically located neuronal loss causes rapid functional decline with comparatively lower overall axonal breakdown. Conversely, head and limb onset may involve earlier degeneration of long‐tract axonal systems, producing higher NfL elevation relative to functional decline. Leg‐onset phenotypes showed slower functional decline, lower NfL levels, and longer survival. Proximal leg onset (O4p) showed significantly lower ALSPR and NfL than distal leg onset and the most favorable survival probability. Overall, proximal limb onset was associated with a less aggressive disease course, indicating that distal–proximal distinctions provide additional prognostic information.

### 4.3 Propagation Phenotypes

Early vertical propagation (PE) emerged as the strongest marker of disease aggressiveness. Patients with PE showed higher ALSPR and elevated NfL, consistent with increased disease activity. Remarkably, among patients with similarly high progression rates, those with PE had higher NfL levels than those with PL, indicating a relative uncoupling of NfL from functional decline (Figure 7). This dissociation may reflect faster functional decline associated with predominantly local spread. Conversely, PE may indicate disease spread along longitudinal neuronal networks, recruiting additional motor territories and producing a greater burden of axonal degeneration and correspondingly higher NfL levels [13, 14]. Propagation phenotype was also a powerful predictor of survival. PE was associated with markedly reduced survival (HR 2.93, *p* < 0.001), exceeding the prognostic effects observed for onset or motor neuron phenotypes. Although spreading mechanisms have long been recognized as essential for the understanding of ALS, propagation has not been systematically operationalized as a time‐defined prognostic phenotype [13, 14, 15, 16, 17]. This binary differentiation of early versus later propagation translates vertical motor symptom spread into a clinically actionable risk marker. The findings suggest that vertical propagation captures a core dimension of ALS aggressiveness not fully reflected by progression rate or NfL levels alone. It may therefore represent a critical prognostic variable in both clinical care and trial design.

### 4.4 Motor Neuron Dysfunction Phenotypes

Motor neuron dysfunction phenotypes showed different patterns of ALS progression, NfL levels, and survival. The balanced UMN/LMN phenotype (M0) exhibited the fastest progression, followed by the dominant LMN (M2d) and dominant UMN (M1d) variants. In contrast, pure UMN or LMN dysfunction (M1p and M2p) was associated with the lowest ALSPR, consistent with previous observations in PLS and PMA [2, 3, 7]. Across the M axis, NfL differed significantly, with highest concentrations in the balanced phenotype (M0) and intermediate levels in dominant phenotypes (M1d and M2d), whereas pure UMN or LMN phenotypes showed lower values. These gradients parallel the phenotype separation in terms of ALSPR and likely reflect the various anatomical extents of UMN and LMN involvement in the motor neuron phenotypes. Survival differed across motor neuron phenotypes, but effect sizes were lower compared with the onset and propagation axis. Dominant UMN dysfunction (M1d) was associated with reduced hazard relative to the balanced phenotype. The absence of events in the pure UMN group (M1p) further underscores its relatively benign short‐term course, consistent with observations in PLS [2, 3, 7]. However, the formal diagnosis of PLS can only be made after an observation period of 48 months [34]. In the present study, pure UMN phenotypes were associated with a favorable prognosis independent of the temporal diagnostic criterion for PLS. No significant differences were observed between balanced and dominant or pure LMN phenotypes. However, when LMN phenotypes (M2d and M2p) were compared with UMN phenotypes (M1d and M1p), the hazard of death was higher in the LMN group (HR 1.96, *p* = 0.006). These results may challenge previous reports suggesting a more favorable prognosis for progressive muscular atrophy (PMA) [2, 3, 35, 36]. However, this analysis combined patients with LMN‐dominant (M2d) and pure LMN (M2p) phenotypes, limiting direct comparison with PMA cohorts. Overall, the findings along the M axis support the notion that LMN involvement may be prognostically more critical. The prognostic relevance of LMN phenotypes may therefore warrant consideration in predictive models and in the design of future ALS clinical trials [1, 4, 32, 37]. M axis classification is time‐dependent, as the relative contribution of UMN and LMN dysfunction may change during disease progression, with increasing dominance of M2d or M2p expected over time. The short observation period precluded a reliable analysis of these transitions, which should be investigated in longer longitudinal studies.

Several limitations warrant consideration. Rare phenotypes, particularly trunk onset (O3) and dissociated motor neuron dysfunction (M3), were expectedly represented by small numbers, limiting statistical power for subgroup analyses. Although each OPM axis was associated with survival in separate models adjusted for age and sex, these analyses do not establish whether its prognostic contribution is independent of the other two axes. The limited number of events and sparse representation of several phenotype combinations precluded robust mutually adjusted or cluster‐based analyses. Future studies using larger datasets and longer follow‐up should determine whether clustering across the three OPM axes can identify reproducible and clinically actionable phenotypes that improve prognostication and patient stratification in clinical trials. Survival was analyzed from study inclusion over a relatively short follow‐up period in a tertiary‐center cohort, making the estimates susceptible to referral and survivorship bias and limiting comparisons with population‐based survival from disease onset. ALS‐OPM phenotyping relied on patient history and neurological examination, which may introduce recall bias and interrater variability. Finally, non‐motor ALS phenotypes, including cognitive and behavioral involvement, were not assessed and may interact with motor phenotypes and biomarker profiles.

In conclusion, ALS has long been described as a clinically heterogeneous disorder. However, this heterogeneity is often treated as noise rather than structured variation reflecting known clinical patterns. The ALS‐OPM framework demonstrates that heterogeneity follows reproducible anatomical determinants that are associated with distinct profiles of ALS progression, NfL elevation and survival. These findings support a model in which the topographical pattern of motor neuron degeneration shapes the clinical trajectory, biomarker expression and survival [13, 14, 15, 16, 17, 38, 39, 40]. Successive ALS‐OPM versions, including the transition from version 3.1 to 3.3, represent incremental refinements of an evolving operational framework, while data generated using version 3.1 remain valid and contribute to its growing evidence base. Incorporating ALS‐OPM phenotypes into clinical research and trial design may reduce heterogeneity, enhance the interpretability of NfL as a biomarker in ALS, and improve prognostic precision, including survival.

## Author Contributions

**Thomas Meyer:** conceptualization, investigation, methodology, validation, visualization, funding acquisition, writing – original draft, supervision, resources, project administration. **Torsten Grehl:** conceptualization, investigation, methodology, validation, writing – review and editing, data curation. **Peggy Schumann:** methodology, validation, visualization, writing – review and editing, software, formal analysis, project administration, data curation, supervision. **Ute Weyen:** investigation, writing – review and editing, validation. **Annekathrin Rödiger:** investigation, validation, writing – review and editing. **Uta Smesny:** investigation, writing – review and editing, validation. **Benjamin Ilse:** investigation, validation, writing – review and editing. **Patrick Weydt:** investigation, validation, writing – review and editing, project administration. **Sarah Bernsen:** investigation, validation, writing – review and editing, project administration. **Rachel Fabian:** investigation, validation, writing – review and editing. **Bettina Göricke:** investigation, validation, writing – review and editing. **Susanne Petri:** investigation, validation, writing – review and editing. **Rea Lumi:** investigation, validation, writing – review and editing. **Bogdan Bjelica:** investigation, validation, writing – review and editing. **Julian Großkreutz:** investigation, validation, writing – review and editing. **Hakan Cetin:** investigation, validation, writing – review and editing, project administration, resources. **Omar Keritam:** writing – review and editing, investigation, validation. **Friedrich Haimberger:** investigation, validation, writing – review and editing. **Catharina Korsukewitz:** investigation, validation, writing – review and editing. **Wolfgang Löscher:** investigation, validation, writing – review and editing. **Zaharias Kohl:** investigation, validation, writing – review and editing. **Martin Regensburger:** investigation, validation, writing – review and editing. **Petra Baum:** investigation, validation, writing – review and editing. **Moritz Metelmann:** investigation, validation, writing – review and editing. **Dagmar Kettemann:** investigation, validation, writing – review and editing. **Jenny Norden:** investigation, validation, writing – review and editing. **Robert Steinbach:** investigation, validation, writing – review and editing. **Péter Körtvélyessy:** investigation, validation, writing – review and editing. **Paul Lingor:** investigation, validation, writing – review and editing, methodology. **Robert McFarlane:** methodology, validation, visualization, writing – review and editing, formal analysis. **Christoph Münch:** investigation, conceptualization, validation, visualization, writing – review and editing, project administration, resources, supervision. **Matthias Boentert:** investigation, conceptualization, methodology, validation, writing – review and editing, formal analysis, project administration, supervision. **André Maier:** conceptualization, investigation, methodology, validation, writing – review and editing, software, formal analysis, project administration, resources.

## Funding

This work was supported by the Boris Canessa ALS Stiftung (Düsseldorf, Germany).

## Conflicts of Interest

T.M. has received grants and research support from AL‐S Pharma, QurAlis, Novartis, and Prilenia, and served as a consultant for Biogen, AbbVie, Voyager Therapeutics, ITF Pharma, Lilly, and Trace Neuroscience outside of the submitted work. T.M. and C.M. are founders and shareholders of the Ambulanzpartner Soziotechnologie APST GmbH, which makes the mobile application “ALS App”. T.G. has received personal fees from ITF Pharma and served on the advisory boards of ITF Pharma outside of the submitted work. J.G. has received grants and research support from AL‐S Pharma, QurAlis, and Prilenia, served as a consultant for QurAlis, ITF Pharma, and Clene Nanomedicine outside of the submitted work. P.W. has served on the advisory boards of Biogen, ITF Pharma, and Novartis outside of the submitted work. S.P. has received speaker fees, nonfinancial support, and research support from Biogen, Roche, AL‐S Pharma, ITF Pharma, and Sanofi, and served on the advisory boards of Biogen, Roche, Zambon, and ITF Pharma outside of the submitted work. P.L. reports grants from the Bundesministerium für Bildung und Forschung and the Deutsche Forschungsgemeinschaft; consulting fees from AbbVie, Amylyx, Bial, Desitin, ITF Pharma, Novartis, Stadapharm, Raya Therapeutic, Woolsey Pharmaceuticals, and Zambon; and is coinventor on a patent for the use of fasudil in amyotrophic lateral sclerosis (EP 2825175 B1, US 9.980,972 B2) outside of the scope of the submitted work. S.P. is an employee of APST, which makes the “ALS App”. The other authors declare no conflicts of interest.

## Supporting information


**Table S1:** Intercenter consistency of ALS‐OPM phenotype frequency distribution.

**Table S2:** ALS progression, propagation phenotypes, and NfL–ALSPR index.

## Data Availability

The data that support the findings of this study are available from the corresponding author upon reasonable request.
