# Supplementary material for: Anatomically Defined Motor Phenotypes Reveal Dissociated Trajectories of Progression, Neurofilament Profiles, and Survival in ALS
Source: Eur J Neurol. 2026 Oct 27;33(11):e70798. doi: 10.1111/ene.70798 (PMC13714081; doi:10.1111/ene.70798)
Supplement: Supplementary file 1 [file ENE-33-e70798-s001.pdf]

Supplementary material

Table S2. ALS progression, propagation phenotypes, and NfL–ALSPR index.

ALS progression	PE, n	PL, n	PE (%)	PL (%)	NfL-ALSPR index, PE	NfL-ALSPR index, PL
Slower	161	483	31.9	80.1	4.862	5.030
Intermediate	183	105	36.3	17.4	4.457	4.517
Faster	160	15	31.7	2.5	4.050	3.891

Data are presented as n, within-phenotype percentage, and NfL-ALSPR index. Percentages were calculated separately within PE (n=504) and PL (n=603). PE, early propagation; PL, later propagation; NfL, neurofilament light chain; ALSPR, ALS progression rate.
