# Supplementary material for: Anatomically Defined Motor Phenotypes Reveal Dissociated Trajectories of Progression, Neurofilament Profiles, and Survival in ALS
Source: Eur J Neurol. 2026 Oct 27;33(11):e70798. doi: 10.1111/ene.70798 (PMC13714081; doi:10.1111/ene.70798)
Supplement: Supplementary file 2 [file ENE-33-e70798-s002.pdf]

Supplementary material

Table S1. Intercenter consistency of ALS-OPM phenotype frequency distribution

ALS-OPM	Patients (n)	Categories	Friedman χ^2	p	Kendall's W	Effect size
Onset (O)	1,123	O1–O4p	48.05	<0.001	0.89	Very strong
Propagation (P)	1,122	PE/PL/PN	13.56	0.001	0.75	Strong
Motor neuron (M)	1,117	M0–M3	34.51	<0.001	0.77	Strong

Center-specific percentages (0–100%) were calculated per axis and restricted to centers with ≥20 patients in the respective axis (9 centers). Friedman tests used df=6 (O axis), df=2 (P axis), and df=5 (M axis). Kendall's W was derived from the Friedman χ^2 statistic.
